# Oxidants and antioxidants relevance in rats' pulmonary induced oxidative stress

**Published:** 2011-08-25

**Authors:** OR Temneanu, C Zamfir, F Eloaie Zugun, E Cojocaru, L Tocan

**Affiliations:** * Department of Histology, ‘Gr. T. Popa’ University of Medicine and Pharmacy, IasiRomania; ** Department of Immunology, ‘Gr. T. Popa’ University of Medicine and Pharmacy, IasiRomania

**Keywords:** oxidative stress, superoxide dismutase, antioxidant defense

## Abstract

**Introduction**: Even if the reactive oxygen species were discovered, described and detailed a long time ago, there is still little data about the mechanisms of oxidative stress, their tissular effects and about an efficient antioxidant strategy, involving animal experimental models. It has been shown that the lung is one of the most exposed organs to the oxidative stress. The particular effects of different types of oxidative stress on lungs were investigated in this experimental study, in order to quantify the intensity and the extent of the pulmonary damage, featuring the antioxidant enzymatic protective role.

**Methods**: The study of lung injury was performed on four distinct groups of Wistar rats: a control group versus a group exposed to continuous light deprivation versus a group exposed to nitrofurantoin versus a group exposed to continuous light deprivation, to nitrofurantoin and vitamin C. Pulmonary samples were taken and treated for microscopic analysis. A qualitative immunohistochemical estimation of pulmonary superoxide dismutase 1(SOD 1) was performed. Blood tests were used in order to reveal the presence and intensity of oxidative stress.

**Results**: Continuous light deprivation and the chronic administration of nitrofurantoin acted as oxidants with a certain involvement in lung damage– vascular and alveolar wall disturbances. Adding an antioxidant, such as vitamin C, considerably improved lung reactivity to oxidative stress.

**Conclusion**: The chronic exposure to oxidants in the induced oxidative stress sustains the development of specific lung alterations. SOD 1 positive reaction underlines the complex enzymatic defense in oxidative stress.

## Background

The extremely large diversity of oxidative stress settlement raised a question about the body's ability to continue detecting a possible variation of its own oxidative status on time [1,2]. The great extent of oxidative stress and its particular impact on lungs promote the interest in developing and outlining new defensive strategies. Reactive oxygen species aggression on lungs will determine (depending mostly on the oxidant stressor type) tissular alterations which will compromise the normal functions of the lungs [3,4].

Every new therapeutic mark intends to improve the defense against oxidative damage. There is a controversy about the existence of a direct correlation between the way the oxidative reactions chain is developed and the tissular injury resulted [5]. Oxidants and antioxidants action in a real time trial, from opposite sides, to interfere with the main oxidative mechanism. If oxidants are present at an elevated rate, they will constantly initiate a chain reaction, which, once started, can evaluate through redox mediated circuits to a high damage potential [6,7]. If antioxidants are mobilized, they work by significantly reducing, preventing or slowing the oxidative damages. They are able to initiate defensive mechanisms, involving the chain breaking or scavenging of initiating radicals [8]. In the light of the role antioxidants play in the development of oxidative stress, evidence is also accumulating that there are specific pathways related to the nature of stress versus antioxidant involvement [9,10].

This experimental study was focused on pulmonary impact of two different types of oxidative stress, finally correlated with the administration of a classic antioxidant, vitamin C. We have also monitored the presence of Cu–Zn superoxide dismutase (SOD 1) in pulmonary tissues and in blood. The targeted involvement of SOD1 can be evaluated as an indispensable input for new therapeutic resources in oxidative stress defense

## Methods

The experiment used four distinct groups, each made up of eight male adult Wistar rats; a control group(1) versus a group exposed to light deprivation(2) versus a group which received nitrofurantoin 125mg/kbw/day(3) versus a group exposed to light deprivation, nitrofurantoin administration 125mg/kbw/day and vitamin C 15mg/100gbw/day(4). After 60 days, at the end of the experiment, we took lung samples, which were prepared for microscopic exam. SOD,GPx and Catalase were measured in blood samples taken in the first, the 30th and the 60th day of the experiment. 

An immunohistochemistry study was realized on formalin–fixed paraffin embedded tissue. Sections of 4μm were cut, dewaxed in xylene and rehydrated in alcohol. Ag retrieval was realized in Citrate buffer (Dako–S 1700), for 25 minutes at 96,5 °C and washed in PBS. After blocking the endogenous peroxidase (3% H2O2) and non–specific binding with Dako S0809, the sections were incubated with adequate dilution (1/200) of Rabbit Anti–Human SOD1 Monoclonal Antibody. Additional control sections were prepared for murine IgG1. Furthermore, we followed the steps for Dako N Vision (Dual link system) and DAB system was used as a chromogen. The sections were finally counterstained with Meyer's Hematoxylin. The seriated slides from control and from the study groups were identically immunostained for a coherent assessment and comparison of results, which were finally codified in estimative scores.

## Results and discussions

The chronic light deprivation, as well as the prolonged administration of nitrofurantoin will produce various degrees of lung damage. The lung inflammatory response is usually followed by a profound epithelial and also endothelial alteration, which will finally generate a compact fibrosis.

**Figure 1:**
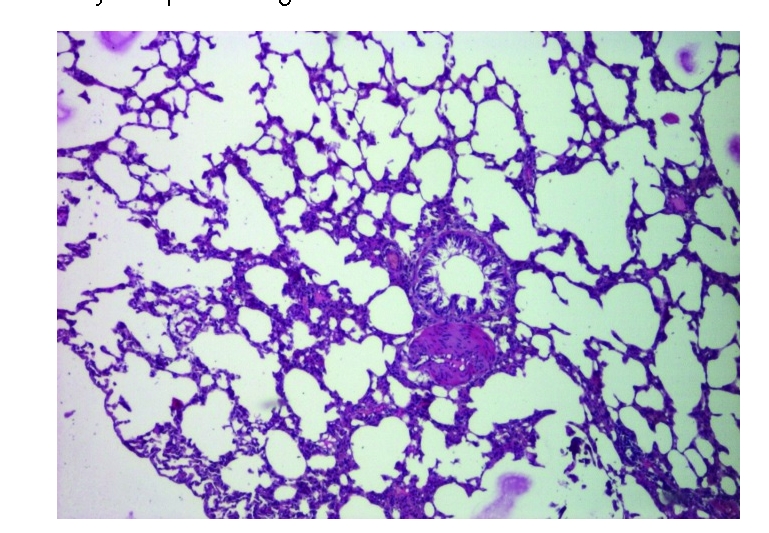
Group 1–control. Normal pulmonary morphology. HE staining X 100

**Figure 2:**
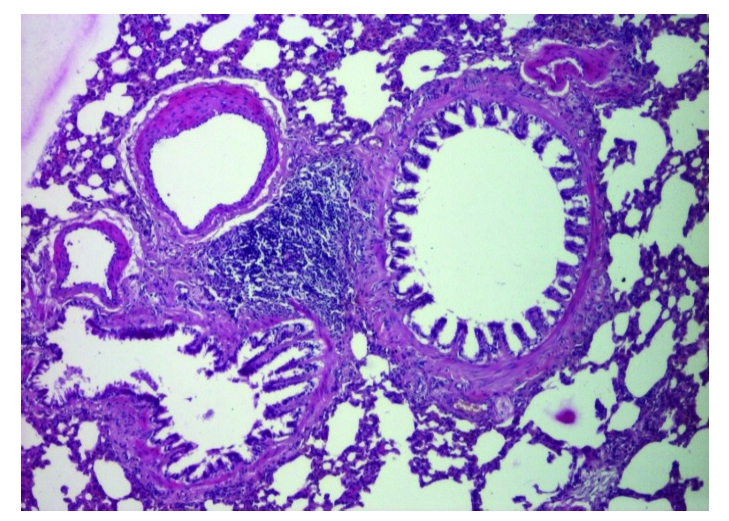
Group 2. Inflammatory areas. HE staining X 200

**Figure 3:**
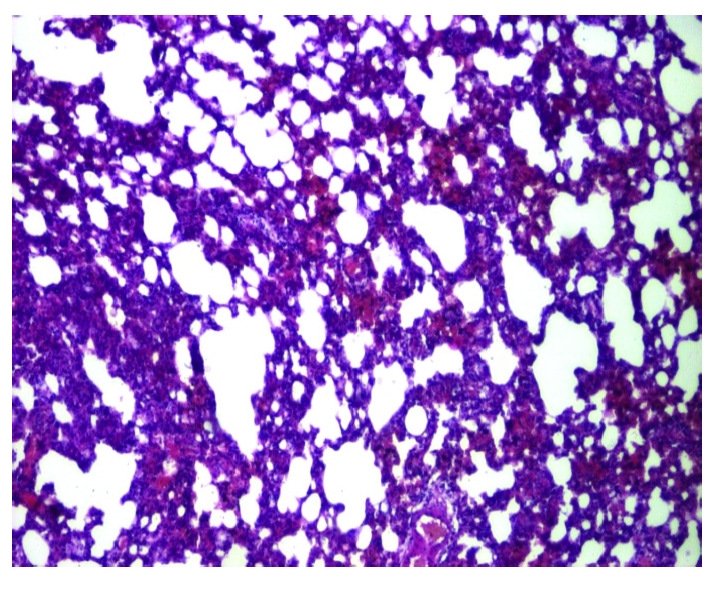
Group 3. Perialveolar fibrosis. HE staining X 100

Both groups of rats exposed to chronobiological or chemical stress will present distinct lung alterations, due to an enhanced, prolonged oxidative stress. Inflammation seems to be scattered in the perialveolar spaces and, usually, it is the first step in the development of progressive thickening of the septal alveolar epithelium and fibrosis (Figure 1, Figure 2, Figure 3)

**Figure 4:**
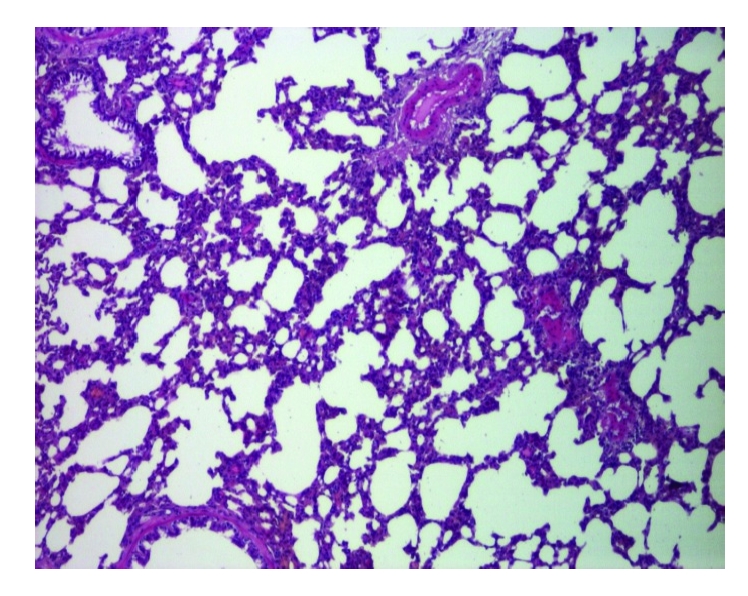
Reduced septal alveolar epithelium thickening. HE staining X 100

The last group of rats was less affected by oxidative stress, due to vitamin C association. (Figure 4)

Blood biochemistry confirmed the existence of the induced oxidative stress, the high level of stress enzymes preventing this fact.

### Immunohistochemical study 

**Figure 5:**
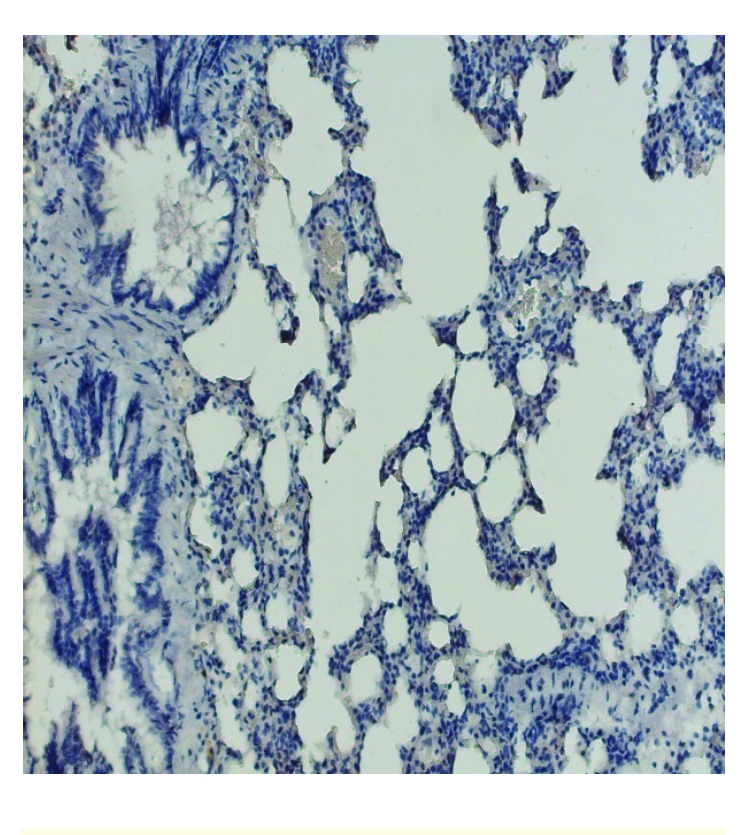
Control group SOD 1–no staining, X100

**Figure 6:**
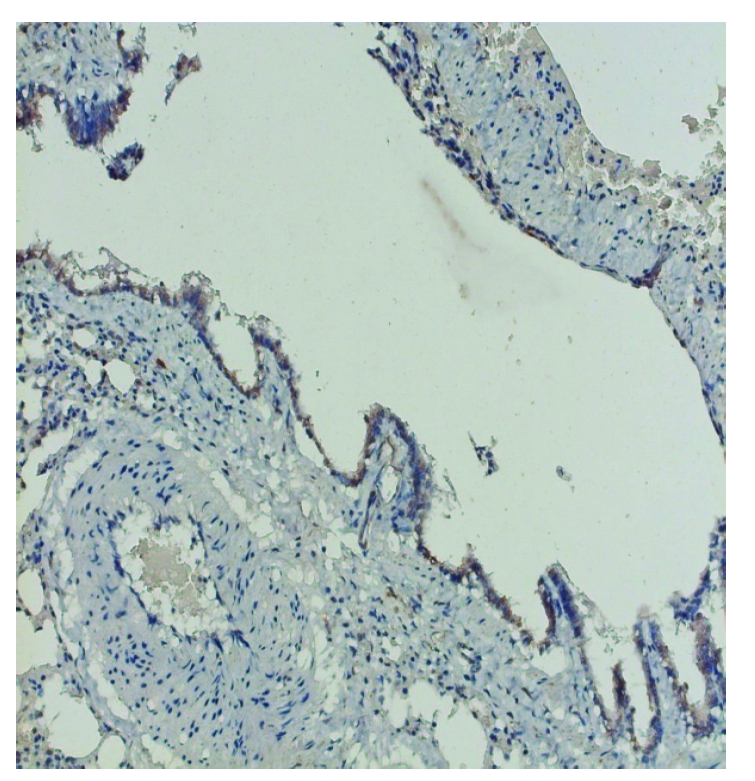
Group 2. SOD1 positive lining bronchiolar epithelial cells, X200

**Figure 7:**
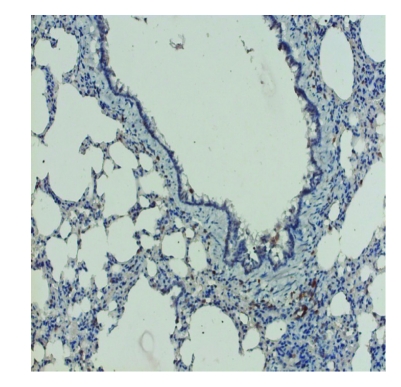
Group 3. SOD1 positive lining alveolar walls and respiratory epithelium, X200

**Figure 8:**
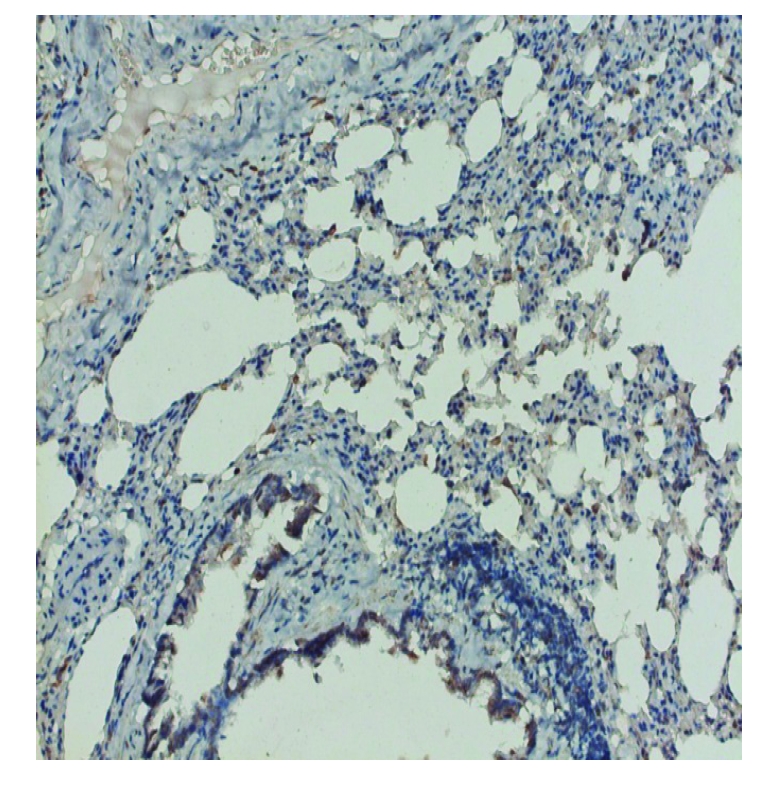
Group 4. SOD 1 moderate staining of bronchiolar epithelium, X200

SOD Immunolocation was positive for the rats exposed to continuous light deprivation or nitrofurantoin, compared with the control group (Figure 5, Figure 6, Figure 7). Moderate expression of SOD1 in the last group of rats suggests that, despite the use of an antioxidant, the intensity of oxidative stress is still up to a high level; a necessary next demand should be to adjust and determine the proper dose of the antioxidant to block the oxidative reactions (Figure 8)

### Statistical analysis of oxidative enzymes

**Table 1:** Descriptive analysis of SOD/group of rats

Lot		R0			R1				R2		
	media	SD	CV%	media	SD	CV%	dif R0	media	SD	CV%
1	183.76	13.06	7.11	183.61	16.99	9.25	–0.08	179.88	10.56	**5.87**	–2.11
2	173.46	16.65	9.60	260.77	23.37	8.96	50.33	269.44	17.08	**6.34**	55.33
3	174.32	18.63	10.69	186.25	15.96	8.57	6.84	246.49	18.95	**7.69**	41.40
4	170.03	14.95	**8.79**	183.25	18.17	9.92	7.77	188.63	20.88	11.07	10.94

**Figure 9:**
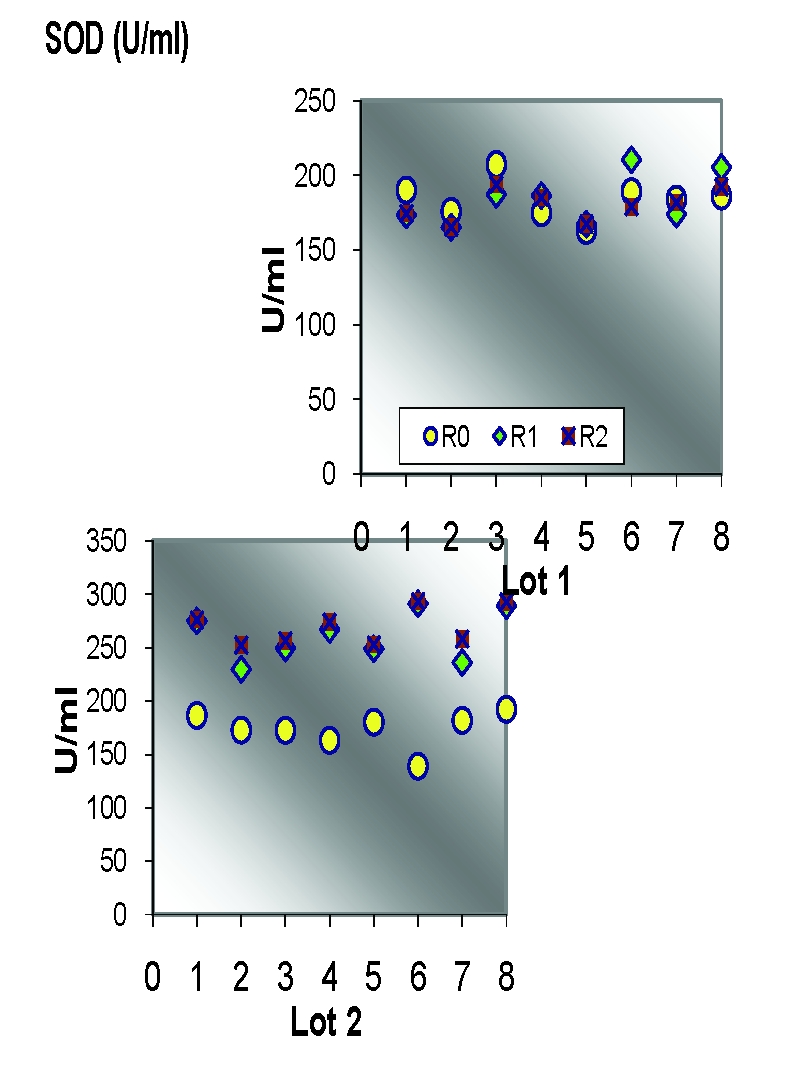
Individual SOD values/study group

**Figure 10:**
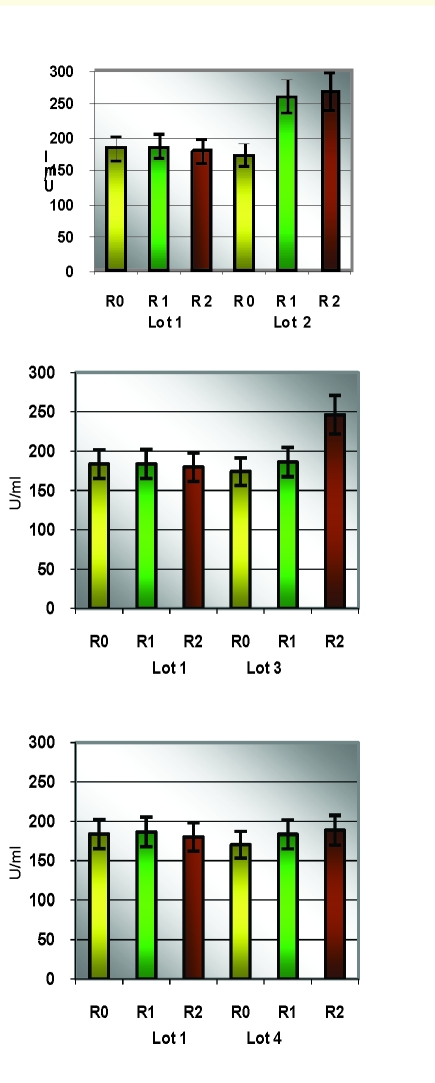
Average SOD values

**Table 2:** SOD comparison between the control group and the groups exposed to stress

Lot 1 vs			R0			R1			R2		
		t		p		t		p		t		p
Lot 2		1.38		p>0.05		7.55		**p<0.001**		12.61		**p<0.001**
Lot 3		1.17		p>0.05		0.32		p>0.05		8.68		**p<0.001**
Lot 4		1.96		p>0.05		0.04		p>0.05		1.06		p>0.05

**Table 3:** Descriptive analysis of catalase/group of rats

		R0			R1				R2		
Lot	media	SD	CV%	media	SD	CV%	dif R0	media	SD	CV%	dif. R0
1	40.72	0.34	**0.83**	40.83	0.43	1.06	0.27	40.80	0.42	1.03	0.21
2	40.88	0.44	1.08	55.97	1.14	2.05	36.92	60.95	0.42	**0.69**	49.12
3	40.92	0.54	1.32	46.10	1.01	2.19	12.66	50.91	0.42	**0.83**	24.43
4	40.70	0.61	1.50	41.85	0.65	1.56	2.83	43.15	0.60	**1.38**	6.02

**Table 4:** Catalase comparison between the control group and the groups exposed to stress

Lot 1 vs			R0			R1			R2		
		t		p		t		p		t		p
Lot 2		0.81		p>0.05		35.15		**p<0.001**		95.95		**p<0.001**
Lot 3		0.89		p>0.05		13.58		**p<0.001**		48.14		**p<0.001**
Lot 4		0.08		p>0.05		3.70		**p<0.002**		9.08		**p<0.001**

**Figure 11:**
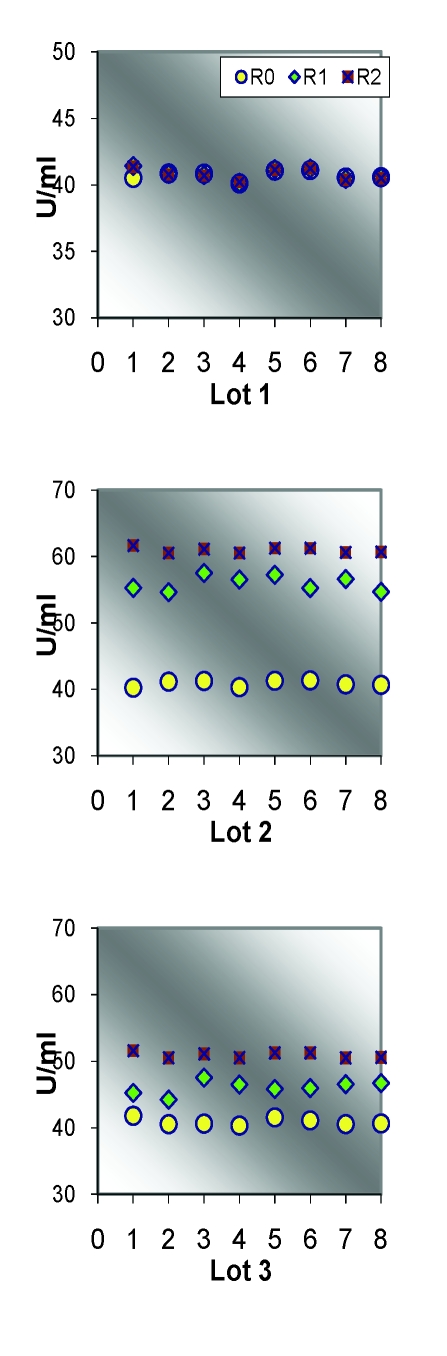
Individual catalase values/study group

**Figure 12:**
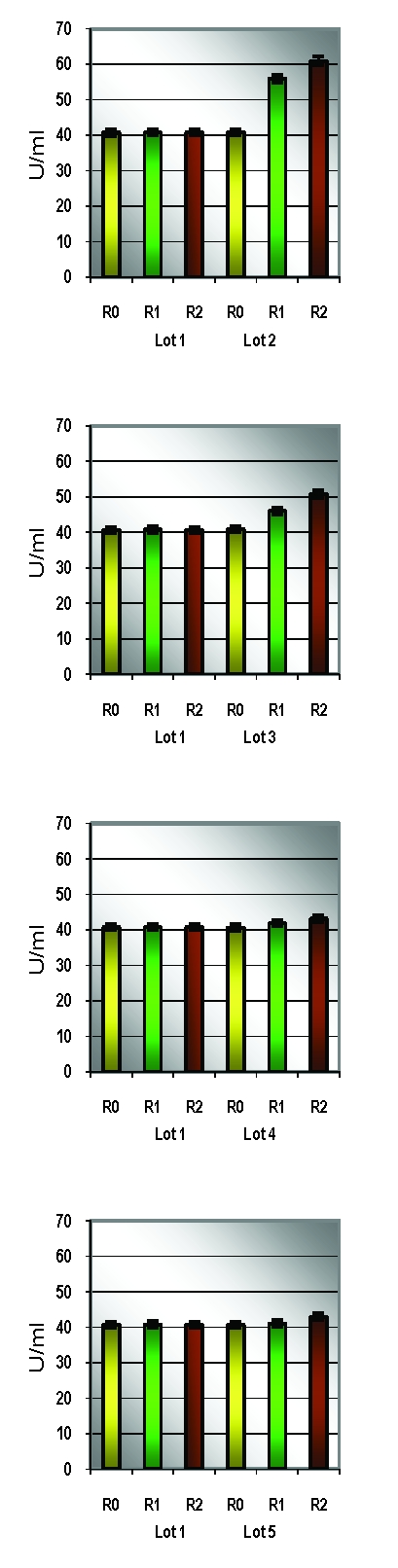
Average catalase values

Statistical analysis of SOD and Catalase (quantified in the beginning, at the middle and at the end of the experimental study) revealed significant correlations between the increasing oxidative stress and the enzymatic levels.

## Discussion

The oxidative stress is known as an undoubtedly factor in determining the impairment of pulmonary structure and function pulmonary responsiveness. The way in which this stress is induced can be very important in the later development of diseases pathogenesis [11,12].

Light deprivation is considered an aggressive form of chronobiologic oxidative stress, which altered not only the lung function and morphology, but also the behavioral status [13]. Disorders of normal circadian rhythm will be directly connected with rats' reactivity to external stimuli. Their biological rhythm is slowed down, promoting a profound apathy [13,14]. Lung histopathological exam revealed specific markers of oxidative stress: inflammation, thickening of alveolar walls and fibrosis.

The third group of rats, presenting nitrofurantoin overexposure, reveals similar lung damage compared with the second group. Prolonged administration of Nitrofurantoin will produce an oxidative aggression of the lungs, chemical stress, acting through oxidative links [15].

The fourth group of rats exposed both to oxidative stress and to antioxidants–vitamin C, revealed only a low but persistent level of oxidative processes, without fibrosis.

Immunohistochemistry was used in order to indicate the distribution of Cu Zn SOD1 after oxidative lung injuries, sustaining the theory that high levels of SOD1 could protect the lung against significant morphological changes due to oxidative stress [16,17]. SOD 1 was decelable in all the study groups–epithelial bronchiolar cells and endothelial cells stain more intensely than alveolar cells in–group two and three. The last group had a moderate SOD1 staining. Vitamin C is an antioxidant, which in normal conditions, attenuates the intensity of oxidative damage [18,19]. The moderate SOD staining reaction for the rats with a double stress exposure confirms the antioxidant capacity of vitamin C.

For the statistic analysis of blood enzymes, which can provide the presence and the intensity of the oxidative stress, we have explored SOD and Catalase. SOD represents one of the most significant defense enzymatic complexes against every oxidative stress, while catalase becomes extremely active in high–level oxidative stress. [20,21] Both of them were gradually raising during the experiment and compared to the control group. Their evolution is correlated with their protective function [22].

## Conclusions

Lung accurately reflects the intensity of induced oxidative stress. The most frequent damages of pulmonary tissue are inflammation, fibrosis and the disruption of vascular walls.

SOD can monitor lung reactivity, but it is very important to estimate in a correct manner the real correlation between the types of stress, the antioxidant involved enzymes and the lung responsiveness. In this context, the animal experimental models offer the possibility of initiating new–targeted antioxidant therapies.
